# The parasitic lifestyle of an archaeal symbiont

**DOI:** 10.1038/s41467-024-49962-y

**Published:** 2024-07-31

**Authors:** Joshua N. Hamm, Yan Liao, Andriko von Kügelgen, Nina Dombrowski, Evan Landers, Christopher Brownlee, Emma M. V. Johansson, Renee M. Whan, Matthew A. B. Baker, Buzz Baum, Tanmay A. M. Bharat, Iain G. Duggin, Anja Spang, Ricardo Cavicchioli

**Affiliations:** 1 School of Biotechnology and Biomolecular Sciences, UNSW Sydney, Sydney, NSW 2052 Australia; 2 Department of Marine Microbiology and Biogeochemistry, Royal Netherlands Institute for Sea Research, Den Hoorn, The Netherlands 1797 SZ,; 3 Australian Institute for Microbiology and Infection, University of Technology Sydney, Ultimo, NSW 2007 Australia; 4 Structural Studies Division, MRC Laboratory of Molecular Biology, Cambridge, CB2 0QH UK; 5 Sir William Dunn School of Pathology, University of Oxford, Oxford, OX1 3RE UK; 6 Biological Resources Imaging Laboratory, Mark Wainwright Analytical Centre, University of New South Wales, Sydney, NSW 2052 Australia; 7 Fluorescence Analysis Facility, Molecular Horizons, University of Wollongong, Keiraville, NSW 2522 Australia; 8 Katharina Gaus Light Microscopy Facility, Mark Wainwright Analytical Centre, The University of New South Wales, Sydney, NSW 2052 Australia; 9 Cell Biology Division, MRC Laboratory of Molecular Biology, Cambridge, CB2 0QH UK; 10 Department of Evolutionary & Population Biology, Institute for Biodiversity and Ecosystem Dynamics (IBED), University of Amsterdam, Amsterdam, The Netherlands

**Keywords:** Archaeal physiology, Archaeal biology, Cellular microbiology

## Abstract

DPANN archaea are a diverse group of microorganisms characterised by small cells and reduced genomes. To date, all cultivated DPANN archaea are ectosymbionts that require direct cell contact with an archaeal host species for growth and survival. However, these interactions and their impact on the host species are poorly understood. Here, we show that a DPANN archaeon (*Candidatus* Nanohaloarchaeum antarcticus) engages in parasitic interactions with its host (*Halorubrum lacusprofundi*) that result in host cell lysis. During these interactions, the nanohaloarchaeon appears to enter, or be engulfed by, the host cell. Our results provide experimental evidence for a predatory-like lifestyle of an archaeon, suggesting that at least some DPANN archaea may have roles in controlling host populations and their ecology.

## Abstract (web summary)

DPANN archaea are a group of microorganisms that require direct cell contact with other archaeal host species for growth. Here, Hamm et al. show that a DPANN archaeon engages in parasitic interactions with its host leading to host cell lysis, thus providing experimental evidence of a predatory-like lifestyle for an archaeon.

## Introduction

DPANN Archaea are extremely diverse and thought to comprise various ectosymbiotic Archaea^1–6^. Initially named after the phyla Diapherotrites, Parvarchaeota, Aenigmarchaeota, Nanoarchaeota, and Nanohaloarchaeota (from which the acronym is derived), the DPANN superphylum is now assumed to include the Woesearchaeota, Huberarchaeota, Pacearchaeota, Mamarchaeota, Micrarchaeota, Altiarchaeota (the only lineage currently predicted to be free-living), and Undinarchaeota^7,8^. DPANN Archaea have been identified across a diverse range of environments on Earth including marine sediments and waters, freshwater ecosystems, hot springs, microbial mats, and in hypersaline systems where Nanohaloarchaeota thrive^2–7,9–12^. The phylogenetic diversity of lineages within the DPANN superphylum seems to rival that of the remainder of the archaeal domain combined, and the DPANN may form one of the earliest diverging branches in the archaeal phylogeny^8,9^. However, cultivation of DPANN has proven difficult, with only three lineages currently represented in published laboratory co-cultures: the Nanoarchaeota^2,13,14^, the Micrarchaeota^15,16^, and the Nanohaloarchaeota^1,17,18^. To date, all successfully cultivated DPANN Archaea are symbionts that require direct cell contact with a host archaeon in order to grow and divide^9^. Most DPANN Archaea lack the capacity to synthesise certain essential molecules including some amino acids, nucleotides, cofactors, and lipids which are presumed to be acquired from their hosts during interactions^9^. However, the processes by which DPANN cells associate with their hosts and proliferate are poorly understood^9^.

One of the best-studied examples is the interaction of the ectoparasitic *Nanoarchaeum equitans* with *Ignicoccus hospitalis*, from which *N. equitans* acquires nucleotides, amino acids, lipids, and cofactors^19^. Microscopy has shown that this is associated with the formation of a narrow membrane channel that appears to connect the cytoplasms of both organisms^19^. Whilst other ‘cytoplasmic bridges’ have been observed between other DPANN and their hosts and are thought to facilitate nutrient transfer^1,16,20^, the proteins forming or catalysing the formation of these structures are unknown^16^. Given that both cells possess complex information processing machinery, including ribosomes, it is not clear how this might be achieved without the two cells exchanging material losing their identity. In addition, many DPANN appear to engage in interactions without forming such structures^15,17^ and the mechanism by which these ectosymbionts acquire nutrients is unclear. Furthermore, the process by which ectosymbionts and their hosts coordinate their cell growth and division cycles are poorly understood with some DPANN (e.g. Nanoarchaeota) remaining predominantly attached to their hosts^2^, while others (e.g. Nanohaloarchaeota) produce large quantities of dissociated cells^1,16^. Thus, much remains to be learned about how DPANN attach, proliferate, detach, and find new hosts.

Recently, the Antarctic DPANN archaeon, *Candidatus* Nanohaloarchaeum antarcticus was discovered to require the haloarchaeon *Halorubrum lacusprofundi* for growth^1^. In contrast to other DPANN–host systems which possess characteristics that limit downstream analyses (e.g. requirements for low pH^15,16^ or high temperature^2,13,14^, sensitivity to physical manipulation^15,16^ or oxygen^2,13,14^, and limited biomass production^15,16^), this system yields large quantities of cells from both organisms, is comparatively simple to manipulate^1^, and the host is genetically tractable^21,22^. Here we report the results of live fluorescence, cryogenic correlative light and electron microscopy, and electron cryotomography demonstrating that during interactions between *Ca*. Nha. antarcticus and *Hrr. lacusprofundi*, host cells accumulate membrane bound structures likely derived from *Ca*. Nha. antarcticus cells within their cytoplasm and undergo lysis in response to symbiont infection.

## Results and discussion

### Dynamics of Ca. Nha. antarcticus—*Hrr. lacusprofundi* interactions

Our enrichment culture of the symbiont *Ca*. Nha. antarcticus and several *Hrr. lacusprofundi* host strains^1^ offers an ideal system for studying archaeal symbiosis. Importantly, these mixed cultures generate large numbers of nanohaloarchaeal cells (so that they make up ~50% of total cells in a co-culture at ~10^8^ cells mL^−1^), which can be isolated, and used to infect a pure culture of a single host strain. In addition to the two species of interest, the enrichment culture also contains a low abundance (<1%) *Natrinema* species, which occupies a similar ecological niche to *Hrr. lacusprofundi* and has proven resistant to attempts to remove it from the culture^1^. To investigate *Ca*. Nha antarcticus—*Hrr. lacusprofundi* interaction dynamics, we used MitoTracker fluorescent dyes^23^ as vital cell stains to identify and track live interactions between *Ca*. Nha. antarcticus and *Hrr. lacusprofundi* strain R1S1^24^ as well as electron microscopy to investigate morphological features.

For this analysis, purified *Ca*. Nha. antarcticus cells were stained with MitoTracker DeepRed (MTDeepRed) followed by incubation with MitoTracker Orange (MTOrange)-stained *Hrr. lacusprofundi*. Live co-cultures of labelled cells were then immobilised and cultured on an agarose gel pad or in a microfluidic flow chamber and imaged using time-lapse fluorescence microscopy, 3D laser scanning confocal microscopy, and 4D (3D time-lapse) live cell imaging. In agreement with previous work on other haloarchaeal species^23^, these Mitotracker dyes are retained by *Hrr. lacusprofundi* cells, and do not affect cell growth rates (Supplementary Figs. 1, 2 and 3), suggesting they are non-toxic.

**Fig. 1:**
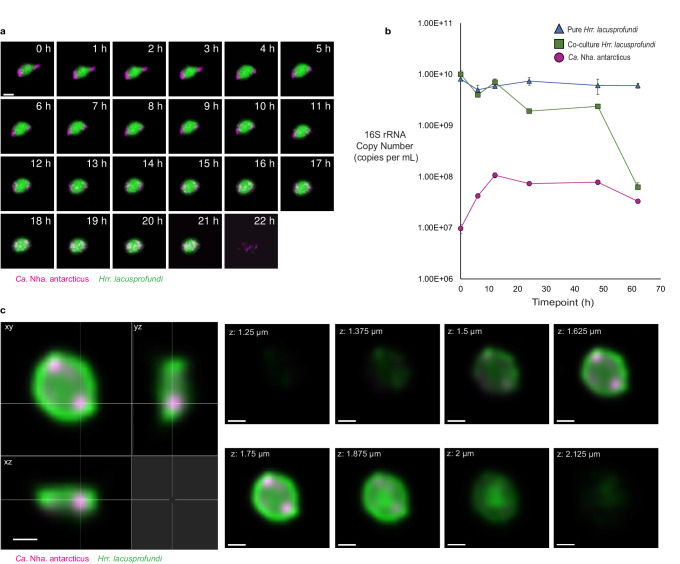
Live fluorescence and qPCR support Ca. Nha. antarcticus entering *Hrr. lacusprofundi* cells and causing lysis. **a** A representative live fluorescence time-lapse series of *Ca*. Nha. antarcticus cells (MitoTracker DeepRed, coloured Magenta) attached to a host *Hrr. lacusprofundi* cell (MitoTracker Orange, coloured Green) (0–9 h), migrating internally (~10–21 h), followed by lysis of the host (22 h). **b**, qPCR quantification of 16S rRNA gene copy numbers from both organisms show active replication of *Ca*. Nha. antarcticus (Magenta circle) during the first 12 h of incubation followed by an ~73% decrease in *Hrr. lacusprofundi* (Green square: Co-culture *Hrr. lacusprofundi*, Blue triangle: Pure *Hrr. lacusprofundi*) 16S rRNA gene copy number between 12 h and 24 h. A second decrease in *Hrr. lacusprofundi* 16S rRNA gene copy number is seen between 48 h and 62 h resulting in a ~ 99% decrease in *Hrr. lacusprofundi* 16S rRNA gene copy number within co-cultures across the entire 62 h incubation compared to ~26% in the pure *Hrr. lacusprofundi* control. Data are presented as the mean value ± the standard deviation across the qPCR reactions (*n* = 3 technical replicates, Source Data are provided as a Source Data file). **c** A 3D confocal orthogonal slice images (left) and z-slices (right) of *Ca*. Nha. antarcticus cells appearing internalised within *Hrr. lacusprofundi* after 10 h incubation. Scale bars: **a** – 1 µm, **c** – 500 nm.

A total of 163 MTOrange-stained *Hrr. lacusprofundi* cells were analysed in detail during two incubations over a period of 24 h on agarose pads. Of these, 132 cells (81%) were observed with one or more MTDeepRed-stained *Ca*. Nha. antarcticus cell(s) attached at the first timepoint imaged (0 h), indicating that attachment predominantly occurred during the initial incubation period (≤ 1 h) prior to commencement of time-lapse imaging (Supplementary Dataset 1). Over time, the fluorescent signal from *Ca*. Nha. antarcticus cells appeared to shift, so that over time more *Ca*. Nha. antarcticus cells appeared to be located within the bounds of their *Hrr. lacusprofundi* host (Fig. 1, Supplementary Fig. 4). Confocal imaging with 3D-orthogonal projection after 10 h incubation showed discrete regions within *Hrr. lacusprofundi* cells positive for MTDeepRed, suggestive of infiltration of the host cytoplasm by *Ca*. Nha. antarcticus (Fig. 1c, Supplementary Fig. 5). The migration of fluorescent signal from nanohaloarchaeal cells inside the boundary of the host appeared to take several hours, but the exact duration varied between different observed interactions (Fig. 1, Supplementary Fig. 4). Once present within the bounds of the *Hrr. lacusprofundi* cell, the area occupied by the *Ca*. Nha. antarcticus dye cell expanded over time (Fig. 1a, Supplementary Figs. 4 and 6).

Over the course of the 24 h incubation period, 27% (36/132) of the *Hrr. lacusprofundi* cells that were observed with attached *Ca*. Nha. antarcticus cell(s) underwent lysis, accounting for 22% (36/163) of total *Hrr. lacusprofundi* cells in co-cultures (Fig. 1a, Supplementary Figs. 4, 5 and 6, Supplementary Dataset 1). Lysis occurred relatively rapidly and was complete within the 30 min time window separating image acquisitions. By contrast, no lysis was observed over periods of up to 70 h in the control samples of pure host cells (Supplementary Fig. 1, Supplementary Dataset 1). Upon lysis, the dye used to label host cells in co-cultures dissipated completely, whereas the label associated with *Ca*. Nha. antarcticus cells remained undimmed (e.g., Fig. 1a, compare 21 h and 22 h). These results are consistent with the survival of *Ca*. Nha. antarcticus cells following host cell lysis.

To investigate whether the timings of events observed in live fluorescence imaging corresponded to observable changes in the 16S rRNA gene copy number of each organism, samples were taken from a co-culture at regular timepoints, and DNA was extracted for qPCR. This revealed a ~10-fold increase in the estimated copy number of *Ca*. Nha. antarcticus 16S rRNA gene copy between 0 and 12 h, indicative of active replication in co-cultures (Fig. 1b). This was accompanied by a moderate decrease in *Hrr. lacusprofundi* 16S rRNA gene copy number (~30%, 0–12 h). Then, between 12 and 24 h, *Hrr. lacusprofundi* 16S rRNA gene copy number decreased by an additional ~73%, leading to a total decrease of ~81% in 16S rRNA gene copy number over the first 24 h. Following this, 16S gene copy numbers stabilised somewhat, before copy number from both organisms displayed a decrease between 48 h and 62 h (*Ca*. Nha. antarcticus: ~42% decrease, *Hrr. lacusprofundi*: ~97% decrease). In total, over the 62 h incubation, *Ca*. Nha. antarcticus 16S rRNA gene copy number increased 3.4-fold, while *Hrr. lacusprofundi* 16S rRNA copy number decreased ~99%. Over the same time period, 16S rRNA gene copy number decreased ~25% in control pure cultures of *Hrr. lacusprofundi*. These data suggest that the interaction of the symbiont with the host is parasitic rather than mutualistic.

**Fig. 2:**
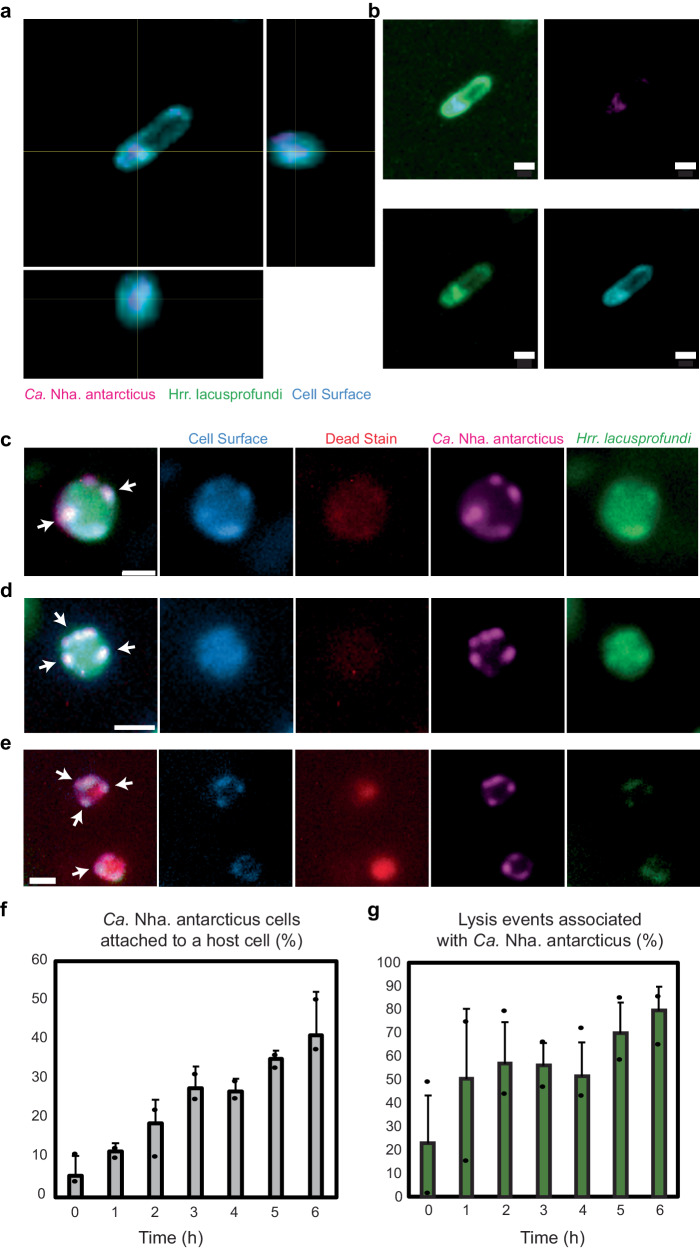
Cell surface stains support the internalisation of *Ca*. Nha. antarcticus material. **a** and **b** Fluorescence micrographs of a co-culture of *Ca*. Nha. antarcticus (Table 1. Nha_FISH_Probe, coloured Magenta), *Hrr. lacusprofundi* (Table 1, Hrr_FISH_Probe, coloured Green), and cell surface (ConA-AF350, coloured Blue). **a** Orthogonal projection of *Hrr. lacusprofundi* cell with signal for the *Ca*. Nha. antarcticus FISH probe inside the bounds of the host cell. **b**, Individual channels and composite image of z-slice from stack used to produce projection in (**a**). **c**–**e** Live fluorescence micrographs taken 6 h post-mixing showing *Ca*. Nha. antarcticus (MitoTracker Green, coloured Magenta) interactions with *Hrr. lacusprofundi* (MitoTracker Orange, coloured Green) including additional stains for cell surface (ConA-AF350, coloured Blue), and cell death (RedDot 2, coloured red). **c** Representative fluorescence micrographs showing *Ca*. Nha. antarcticus cells (MitoTracker Green, coloured Magenta) attached to the surface of *Hrr. lacusprofundi* (MitoTracker Orange, coloured Green). Cell surface staining (ConA-AF350, coloured Blue) shows foci corresponding to regions where *Ca*. Nha. antarcticus was attached to the host cell. No signs of lysis were detected by a dead cell stain (RedDot 2, coloured Red). **d** Representative live fluorescence micrographs showing *Ca*. Nha. antarcticus cells (stained with MitoTracker Green, represented Magenta) that appear internalised within *Hrr. lacusprofundi* cells (stained with MitoTracker Orange, represented Green). Cell surface staining (Concanavalin A, represented Blue) does not show foci corresponding to *Ca*. Nha. antarcticus cells, indicating the surface of the symbiont is inaccessible to the dye. No signs of lysis are evident from inclusion of a dead stain (RedDot 2, represented Red). **e** Representative fluorescence of *Hrr. lacusprofundi* (MitoTracker Orange, coloured Green) lysis events associated with *Ca*. Nha. antarcticus (MitoTracker Green, coloured Magenta). Lysis is indicated by positive fluorescence for RedDot 2 (coloured Red) and is associated with loss of MitoTracker Orange signal from the host cell while the *Ca*. Nha. antarcticus cells remain intact and positive for both MitoTracker Green and the cell surface stain (Con-AF350A, coloured Blue). Quantitative data for (**f**) lysis and (**g**) attachment events over short-term incubations. Data show (**f**) percentage of lysis events associated with a *Ca*. Nha. antarcticus cell and (**g**) the percentage of *Ca*. Nha. antarcticus cells attached to host cells over the course of a time series (0–6 h). Data show average number of events across triplicate experiments, and error bars represent standard deviation as summarised in Supplementary Dataset 2. Arrows: examples of *Ca*. Nha. antarcticus cells; Scale bars: **a,** **b**: 1 µm, **c**–**e**: 500 nm.

To assess replicability and specificity of apparent internalisation, 16S rRNA targeted FISH microscopy with addition of a lectin cell surface stain (Concanavalin A conjugated with Alexa Fluor 350 (ConA-AF350)) was conducted on samples of co-cultures incubated for 16 h (Fig. 2a, b). A Z-stack of a *Hrr. lacusprofundi* cell co-fluorescent for the *Ca*. Nha. antarcticus 16S rRNA probe shows nanohaloarchaeal 16S rRNA signal localised within the host cell, and within the region bounded by ConA-AF350. These data support the idea that the cytoplasmic contents of these nanohaloarchaeal cells have entered the host (Fig. 2a, b). To further test whether the translocation of the *Ca*. Nha. antarcticus contents into the *Hrr. lacusprofundi* cells corresponded to the complete internalisation within a live host, ConA-AF350 was used together with a live-cell-impermeable stain (RedDot 2) to both label surface-bound nanohaloarchaeal cells and to assess loss of host cell membrane integrity, respectively. As expected, ConA-AF350 added to co-cultures labelled *Ca*. Nha. antarcticus cells that were attached to the surface of *Hrr. lacusprofundi* (Fig. 2c, Supplementary Fig. 7). By contrast, when ConA-AF350 was added to co-cultures at later time-points (6 h), many foci positive for the *Ca*. Nha. antarcticus dye did not appear positive for ConA-AF350 (Fig. 2d, Supplementary Fig. 8), consistent with their complete internalisation within host cells. At the same time, the absence of RedDot 2 staining indicated that host cells remained intact during the internalisation process (Fig. 2d, Supplementary Fig. 8). Conversely, host cells that were inferred to have lysed via the loss of MitoTracker Orange signal stained positive for RedDot 2 as expected (Fig. 2e, Supplementary Fig. 8). Over the course of 0–6 h incubations, the proportion of *Hrr. lacusprofundi* lysis events associated with *Ca*. Nha. antarcticus cells increased from ~23% to ~80%, while the proportion of nanohaloarchaeal cells attached to a host cell increased from ~6% to ~41% (Fig. 2f, g, Supplementary Dataset 2). Throughout, *Ca*. Nha. antarcticus cells associated with lysed *Hrr. lacusprofundi* cells labelled positive for both MitoTracker and Concanavalin A stains (Fig. 2e, Supplementary Fig. 9).

To complement this analysis, similar experiments were performed using continuous liquid flow culture (in a microfluidics system) to assess the interactions of immobilised, MTOrange stained *Hrr. lacusprofundi* R1S1 cells (0.7–1.1 μm trap height) with MTDeepRed stained, FACS-purified, *Ca*. Nha. antarcticus cells (Supplementary Fig. 6, Supplementary Movie 1). As with the agarose pad experiments, *Ca*. Nha. antarcticus cell(s) attached to *Hrr. lacusprofundi* cells before the first images could be observed. Again, over a 2–23 h time-period, the presence of the internalised *Ca*. Nha. antarcticus MTDeepRed signal was associated with decreased signal intensity and increased area within the host (Supplementary Fig. 6). It was notable that during the time course, 360 *Hrr. lacusprofundi* cells lysed in the infected culture (56%), whereas no lysis occurred in the uninfected and unstained control (407 cells), and only two lysis events occurred in the uninfected and stained control (654 cells) (Supplementary Fig. 2 and 6, Supplementary Dataset 1). Taken together, these agarose pad and microfluidic experiments demonstrate that *Ca*. Nha. antarcticus cells induce lysis of their hosts (22–56% of total *Hrr. lacusprofundi* cells in the infected cultures were lysed versus ~0% in the control (Supplementary Dataset 1)).

Experiments were also performed to investigate the effect of *Ca*. Nha. antarcticus on the morphology of *Hrr. lacusprofundi*, which includes rods, disks, and coccoid cells (Supplementary Fig. 2, Supplementary Dataset 1 and ref. ^25,26^). After co-incubation with MTDeepRed-stained *Ca*. Nha. antarcticus cells, 34% rod-shaped MTOrange-stained *Hrr. lacusprofundi* cells (on agarose pad) had become more rounded (Supplementary Figs. 4 and 10). This morphological change was not seen for control *Hrr. lacusprofundi* cells (Supplementary Figs. 1 and 10, Supplementary Dataset 1). A higher proportion of such morphological changes of co-cultured *Hrr. lacusprofundi* compared to pure culture was also seen with the microfluidics experiments (Supplementary Dataset 1). These results suggest that the association of the two species impacts the structure of the host cell envelope or the arrangement of S-layer proteins of the host cell.

### Structural features of the Ca. Nha. antarcticus symbiosis

**Fig. 3:**
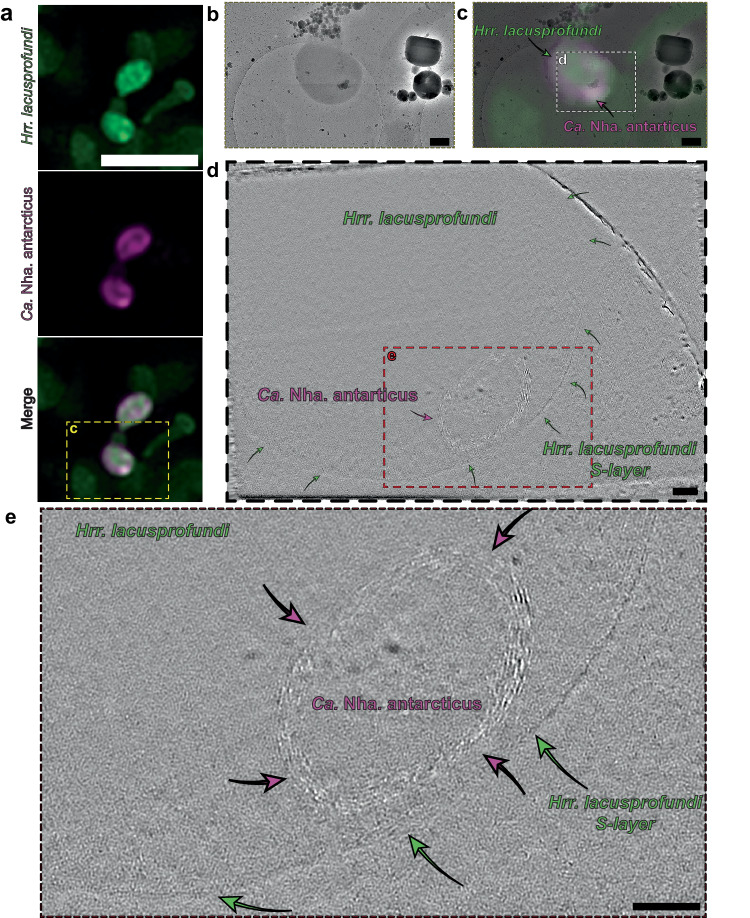
Cryo-correlative light and electron microscopy of an internal structure within a *Hrr.lacusprofundi* cell from a *Ca.* Nha. antarcticus – *Hrr. lacusprofundi* co-culture. **a** Cryo-fluorescence microscopy images show a *Hrr. lacusprofundi* cell (stained with MitoTracker Green, coloured green) with signal consistent with internalisation of *Ca*. Nha. antarcticus cytoplasm (stained with MitoTracker DeepRed, coloured Magenta). **b** Cryo-TEM micrograph of the same cells shown in (**a**) used for identification of regions for tomography. **c** Overlay of cryo-fluorescence and cryo-TEM images. Z-slices from tomogram of internalised structure showing **d** full field of view and **e** internal structure. The cell envelope of the internal structure appears to possess multiple additional layers compared to non-internalised nanohaloarchaeal cells (Supplementary Fig. 14a–d). Due to logistics of equipment access these experiments were performed once. Scale bars: **a** 5 µm, **b**, **c** 500 nm, **d**, **e** 100 nm.

To investigate the structural features of cells in which nanohaloarchaeal cytoplasmic contents labelled with MTDeepRed were seen within the bounds of a host *Hrr. lacusprofundi* cell, we performed cryo-correlated light and electron microscopy (cryo-CLEM) followed by electron cryo-tomography (cryo-ET). Cells were fluorescently labelled as before and incubated together for 16 h to enable attachment and invasion prior to vitrification by plunge-freezing and imaging. This timepoint was chosen to maximise the chances of observing cells within their hosts. When imaging the fluorescent stain using CLEM, we looked for *Hrr. lacusprofundi* cells that were co-labelled for the MTDeepRed used to stain *Ca*. Nha. antarcticus. This identified examples in which MTDeepRed fluorescence was confined to discrete regions of the host cell or was present throughout the host cell (Fig. 3, Supplementary Fig. 11, Supplementary Movies 2 and 3). Cryo-ET of the subset of cells that possessed localised fluorescent signals from MTDeepRed revealed internal membranous structures at locations where MTDeepRed signal was present (Fig. 3). Similar structures could also be identified in cells with diffuse MTDeepRed signal (Supplementary Fig. 11).

To investigate the ultrastructure of *Hrr. lacusprofundi* and *Ca*. Nha. antarcticus in greater detail, higher quality three-dimensional images of *Hrr. lacusprofundi* and *Ca*. Nha. antarcticus cells were acquired for both pure samples and co-cultures using cryo-ET. *Ca*. Nha. antarcticus cells observed in pure samples and in co-cultures that were external to *Hrr. lacusprofundi* cells possessed structures resembling a classical archaeal cell envelope structure with a single lipid bilayer and S-layer. Consistent with the cryo-CLEM data, internal membrane-bound structures (~80–250 nm diameter) were observed in several cryo-ET samples of *Hrr. lacusprofundi* cells incubated with *Ca*. Nha. antarcticus cells (Supplementary Figs. 12 and 13, Supplementary Movies 4−8). In some cases, internal structures were visible in intact host cells as visualised by scanning in z (Supplementary Figs. 12a–c, 13c, d)—consistent with the idea that the structures formed within *Hrr. lacusprofundi* cells can occur without inducing host cell lysis. At the same time, internal structures were also seen in cells that appeared damaged or with a disrupted outer membrane (Supplementary Figs. 12d–f, 13a, b). In both cases, the internalised structures were highly radiation sensitive, similar to the fluorescently labelled structures observed with cryo-CLEM, limiting the achievable resolution of the images. Nevertheless, in many instances, the internal membrane-bound structures seen in co-cultures had a surface that exhibited a repeating pattern^16,27^ characteristic of an S-layer (Supplementary Fig. 12b). Archaea and Bacteria are known to possess several mechanisms to prevent S-layer proteins assembling in the cytoplasm, indicating that these repeating structures are unlikely to constitute the host S-layer^28–30^. The presence of internal membranes and a putative S-layer within infected hosts suggests that these features may represent intact *Ca*. Nha. antarcticus cells or material derived from *Ca*. Nha. antarcticus cells (see Supplementary Discussion: Internal Membrane-bound Structures).

In pure *Ca*. Nha. antarcticus samples, cells exhibited bulges within the membrane and cytoplasmic structures (Supplementary Fig 14a–d, Supplementary Movies 9−12). In appearance, these cytoplasmic structures in *Ca*. Nha. antarcticus, which possess a surface monolayer surrounding a higher electron density core and uniform texture, resemble polyhydroxyalkanoate-like (PHA-like) granules previously identified in *Hrr. lacusprofundi*^31^. The bulges within the *Ca*. Nha. antarcticus membrane resemble lipid droplets^32^. It is notable that similar structures were also observed in the membranes of *Hrr. lacusprofundi* cells interacting with *Ca*. Nha. antarcticus cells (Supplementary Fig. 12d–f and 14e–h, Supplementary Movies 6, 13, 14) but were not observed in the membranes of uninfected *Hrr. lacusprofundi* cells (Supplementary Fig. 14i–k, Supplementary Movies 15−17), suggesting that the Nanohaloarchaeota play a role in inducing their formation. This is potentially significant as *Ca*. Nha. antarcticus lacks identifiable genes for both lipid biosynthesis and metabolism^1^ and must therefore acquire bulk lipids from the host to survive.

We also used Cryo-ET to examine the contact sites between *Ca*. Nha. antarcticus and *Hrr. lacusprofundi* cells prior to invasion (Supplementary Fig. 14e–h, Supplementary Movies 13 and 14). In some cases, these images suggested disruption of the two cell membranes and the opening of a cytoplasmic channel—similar to the structure of the interaction interface previously reported for *N. equitans* and *I. hospitalis* cells^19^. In these cases, a gap is visible in the S-layers of both organisms at the interaction site (Supplementary Fig. 14e–h, Supplementary Movies 13 and 14). In all cases, despite the close physical association of the two cells, this region of close membrane apposition was limited to a section of ~15–20 nm in width.

### Candidate genes mediating interactions

**Fig. 4:**
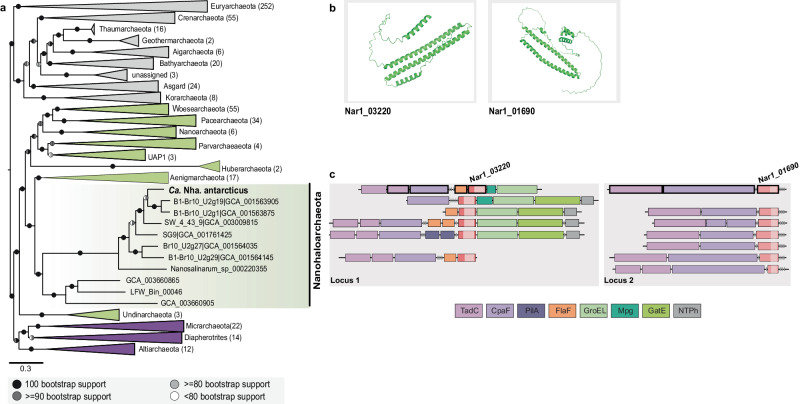
Conservation of loci encoding CCP genes in Nanohaloarchaeota. **a** A maximum-likelihood phylogenetic tree based on 51 marker proteins and 569 archaeal species. The alignment was trimmed with BMGE^61^ (alignment length, 11399 aa). Tree was inferred in IQ-TREE^81^ with the LG + C20 + F + R model with an ultrafast bootstrap approximation (left half of bootstrap symbol) and SH-like approximate likelihood test (right half of bootstrap symbol), each run with 1000 replicates (see key for shading indicating bootstrap support). The tree was artificially rooted between DPANN Archaea (cluster 1 DPANN in dark purple, cluster 2 DPANN in green) and all other Archaea (shaded in grey). The number of species represented in each clade is shown in parentheses after the taxonomic name of the clade. Scale bar: average number of substitutions per site. **b** OmegaFold predicted coiled-coil structures of both the *Ca*. Nha. antarcticus locus 2 CCPs (NAR1_03220 and NAR1_01690). **c** The two Cluster 2 DPANN loci are shown aligned to the Nanohaloarchaeota sequences in the phylogenetic tree. *Ca*. Nha. antarcticus proteins identified in proteomic data are highlighted (bold outline). The type-IV filament proteins encoded in each locus (CpaF, pilus assembly ATPase; TadC, membrane assembly platform) or just Locus 1 (FlaF and PilA, filament proteins) are shown. Other proteins encoded in Locus 1 are Mpg (3-methyladenine DNA glycosylase), GroEL (chaperonin), GatE (Archaeal Glu-tRNA (Gln) amidotransferase subunit E) and NTPhyd (P-loop containing nucleoside triphosphate hydrolase). The gene-locus images were manually generated and loci were only included if they had putative flagella or pili genes up- or downstream of the CCP genes.

In an effort to better understand the molecular mechanisms allowing *Ca*. Nha. antarcticus to engage in this peculiar case of archaeal parasitism, in this study we also took a closer look at the *Ca*. Nha. antarcticus genome. Type IV pili are believed to play an important role in the lifestyles of *Bdellovibrio bacteriovorus*^33^, *Candidatus* Vampirococcus lugosii^34^, *Ca*. Saccharibacteria TM7i^35^, and may also facilitate interactions between *Ca*. Nha. antarcticus and *Hrr. lacusprofundi*. Analysis of a set of 569 representative archaeal genomes revealed the presence of two conserved loci encoding Type-IV pilus homologues across multiple cluster 2 DPANN^8^ lineages (Nanohaloarchaeota, Woesearchaeota, Pacearchaeota, Nanoarchaeota, and Aenigmarchaeota) as well as Undinarchaeota (Fig. 4). In addition to Type-IV pilus genes, these loci also encode proteins with coiled-coil domains predicted to structurally resemble viral attachment proteins (sigma-1 protein: PDB_ID 6GAO, Fig. 4, Supplementary Figs. 15–23). Since similar loci are also present in a cultivated species of Nanoarchaeota (*Ca*. Nanoclepta minutus^13^), which has not been reported to induce similar internal structures as *Ca*. Nha. antarcticus, it remains to be investigated whether they are involved in forming the structures observed in the system studied here. Previously generated proteomics data^1^ confirmed several proteins within the *Ca*. Nha. antarcticus loci are actively synthesised, including the coiled-coil domain containing proteins. In addition to the Type-IV pilus-like loci, comparisons of the genetic content between *Ca*. Nha. antarcticus and *Ca*. Nanohalobium constans^17^ (a cultivated nanohaloarchaeon that was not reported to invade host cells) revealed that *Ca*. Nha. antarcticus encodes proteins that structurally resemble autolysins, bacteriocins, and phage cell-puncturing proteins (Supplementary Discussion), which are absent from the *Ca*. Nanohalobium constans genome, suggesting the possibility that they may support predation of the host.

### Ca. Nha. antarcticus is a parasitic archaeon

Our data demonstrates that the relationship between *Ca*. Nha. antarcticus and *Hrr. lacusprofundi* is parasitic, with interactions between the two organisms leading to lysis of a large proportion of host cells. This likely explains why co-cultures of the two organisms cannot be stably maintained^1^. Several lines of evidence also show that either entire nanohaloarchaeal cells or nanohaloarchaeal cytoplasmic contents enter the host, prior to host cell lysis. First, fluorescence microscopy approaches demonstrate that initial attachment of nanohaloarchaeal cells is followed by internalisation of the nanohaloarchaeal MTDeepRed signal, so that the signal from the nanohaloarchaeal cell eventually appears diffuse and fully encapsulated within the host cell (Fig. 1, Supplementary Fig. 4). Second, whereas externally attached nanohaloarchaeal cells can be labelled using a surface dye (ConA), the surfaces of nanohaloarchaeal cells that appear internalised based on the MTDeepRed signal are inaccessible to an external dye, (Fig. 2, Supplementary Fig. 8). Finally, using cryo-CLEM, we were able to visualise internal, membrane bound structures within infected *Hrr. lacusprofundi* cells at locations that were correlated with the presence of fluorescently labelled nanohaloarchaeal cells (Fig. 3, Supplementary Fig. 11). Taken together, these data suggest that *Ca*. Nha. antarcticus cells may invade the host cytoplasm during infection.

It should be noted that many of the internalised structures observed were smaller than free-living nanohaloarchaeal cells. Thus, it is possible that these internal structures arise from surface-bound nanohaloarchaeal cells, rather than from their complete internalisation. In the event these structures represent bona fide live internalised nanohaloarchaeal cells, the internalisation process may facilitate the acquisition of essential nutrients, including the lipids required for membrane formation and those forming lipid droplets in free *Ca*. Nha. antarcticus cells. Given the eventual loss of host cell integrity following infection, the internalised *Ca*. Nha. antarcticus cells could be released via host cell lysis. In some cases, however, Cryo-ET images of internal structures were suggestive of a multi-layered internalised envelope (Fig. 3), indicating that part of the host surface may be internalised as well as the symbiont cell—perhaps in a process akin to endocytosis, or via internal vesiculation like that observed in L-form bacteria^36^. While internalisation via an endocytosis-like event could enable a nanohaloarchaeal cell to enter a host without bursting it more easily than alternative entry mechanisms, it is possible that the apparent multi-layered envelopes observed represent artifacts of electron damage, which can affect tomographic reconstructions (Fig. 3). The dose sensitivity of the sample limited our capacity to determine which of these possible explanations is most likely. Thus, further work will be necessary to confirm the putative internalisation of *Ca*. Nha. antarcticus and determine how this may be achieved.

The observed activity of *Ca*. Nha. antarcticus shows similarities to the recently reported lifestyle for *Ca*. V. lugosii, a CPR bacterium recently reported to prey on a gammproteobacterium^34^. However, *Ca*. V. lugosii does not appear to invade its host cells but remains in an ectosymbiotic state^34^. The apparent internalisation of nanohaloarchaeal cytoplasmic contents (either via intact cells or vesicles) also bears some similarities to viruses and some bacterial predators, most notably *B. bacteriovorus*^33^. While there are examples of archaeal endosymbionts of eukaryotes (e.g. methanogenic protist endosymbionts^37,38^), Archaea have not previously been shown to enter other archaeal cells, to host intracellular symbionts, or to induce internal vesiculation in symbiotic partners.

In describing a parasitic DPANN archaeon whose interactions with its host results in host cell lysis, this work adds to a growing number of examples of species across both Bacteria and Archaea with the capacity to impact community structure through host species predation^34,39^. It is unclear how widespread such parasitic lifestyles are amongst DPANN Archaea, as the majority of DPANN are uncultivated^8^ and the factors that influence growth of DPANN remain enigmatic. However, the observations we describe in this paper illustrate the potential capacity of certain DPANN Archaea to contribute to nutrient cycling through lysis of host cells, similar to viral predation in the top-down control of the food web in Antarctic aquatic systems^40^. The lysis of host cells and release of organic material into the environment by *Ca*. Nha. antarcticus is likely to increase supply of organic and inorganic nutrients to the wider microbial community. This may, in turn, stimulate growth of diverse members of the community and prevent sequestration of nutrients within host cells. In this way, *Ca*. Nha. antarcticus is likely to contribute to the recycling of nutrients in the three haloarchaeal-dominated, hypersaline lakes that it is known to colonise^1^. Considering that it has been suggested that DPANN Archaea may associate not only with other archaea but also with Bacteria^7,9,41,42^, the capacity of some representatives of the DPANN Archaea to behave in such a predatory manner could have implications for microbial food web dynamics across the globe and may necessitate a re-evaluation of their functional importance and ecological roles.

## Methods

### Production of nanohaloarchaeal cells

Purified *Ca*. Nha. antarcticus cells were sourced from an enrichment culture (Nha-CHl) grown at 18 °C by FACS (Supplementary Fig. 24) as previously described^1^ or through filtration. To acquire *Ca*. Nha. antarcticus through filtration 10 mL of the Nha-CHl culture was first filtered three times through a 0.8 µm pore size cellulose acetate syringe filter and then subsequently filtered five times through a 0.22 µm pore size cellulose acetate syringe filter. The resulting filtrate was centrifuged at 20,000 *g* for 10 min and the cell pellet was resuspended in 1 mL of fresh DBCM2^1^. To confirm purity of filtered cells, aliquots were spot plated on DBCM2 agar and incubated for 2 months at 30 °C. Absence of growth indicated filtration had successfully removed *Hrr. lacusprofundi* cells from the sample. *Hrr. lacusprofundi* strain R1S1^43^ cells were grown as previously described for strain ACAM34^1^, and after two weeks growth, incubated with FACS-purified *Ca*. Nha. antarcticus cells.

### Live fluorescence microscopy

MitoTracker dye (1:1000 dilution; 1 μM final centration) was added to 500 μL of Ca. Nha. antarcticus sorted cells (~ 2 × 10^7 ^mL^−1^; MitoTracker Deep Red FM) or *Hrr. lacusprofundi* cells (~3 × 10^8 ^mL^−1^; MitoTracker Orange CMTMRos)^23^. Cells were maintained at 30 °C with static incubation for 1 h. The dye was washed out three times with fresh DBCM2 media^1^ via centrifugation after staining and resuspended in 50 μL (*Ca*. Nha. antarcticus cells) or 250 μL (*Hrr. lacusprofundi*) of DBCM2 media. Resuspended *Hrr. lacusprofundi* (2 μl) and *Ca*. Nha. antarcticus (4 μL) cells were mixed prior to use. For live-cell fluorescent microscopy imaging, 3 μL of mixed cells was placed on a ~1 mm thick agarose pad (0.3% w/v agarose and containing the full media requirements for DBCM2 media), that had been prepared on an 8 mm diameter #1.5 circular glass coverslip (World Precision Instruments, Inc). The coverslip-pad-cell sample assembly was placed inverted onto the base of a 35 mm #1.5 FluoroDish (WPI)^43^. The pre-warmed (30 °C) liquid DBCM2 medium (4 mL) was gently applied to cover the pad assembly in the FluoroDish. The lid was applied to avoid evaporation and the dish was incubated on the microscope stage (at 30 °C) for imaging. The initial stages of microscope setup for obtaining images of multiple, individual cells took ~1 h, meaning that cells had the opportunity to interact prior to the initiation (*t*_0_) of the time course. Time-lapse fluorescence imaging was performed at 30 °C on a Nikon Ti-E-Perfect Focus microscope with DS-Qi2 camera and a × 100 Oil Plan NA 1.45 objective using a TRITC filter (Ex: 535/36 nm; Em: 590/34 nm) for the MitoTracker Orange fluorescence signal, and a Cy5 filter (Ex: 645/30 nm; Em: 660/40 nm) for the MitoTracker Deep Red fluorescence signal. Z-stack imaging was performed on a confocal laser scanning Nikon A1 microscope with A1-DUG GaAsP Multi Detector Unit (hybrid 4-channel detector: 2 GaAsP PMTs + 2 normal PMTs) at 30 °C using a Plan Fluor 100 × Oil objective (z-axis step 0.125 μm) with the TRITC filter (Ex: GaAsP 561 nm; Em: 595/50 nm) and Alx647 channel (Ex: PMT, 637.4 nm; Em: 700/75 nm), or on a DeltaVision Elite microscope at 30 °C using a 100 × Oil NA 1.4 objective (Z-axis step 0.2 μm or 0.5 μm) with the TRITC (Ex: 531-565 nm; Em: 573-611 nm) and Cy5 (Ex: 619–649 nm; Em: 654–700 nm) filters. The imaging data were processed for deconvolution and bleach correction as stated in the figure legend. The processed Z-stack data were re-constructed for 3D ‘orthogonal’ slice projection analyses using the Imaris software package (Bitplane AG, Zurich, Switzerland).

To determine whether translocation of the *Ca*. Nha. antarcticus stain into *Hrr. lacusprofundi* R1S1 cells corresponded to internalisation or invagination, cells were stained with either MitoTracker Orange (*Hrr. lacusprofundi* R1S1) or MitoTracker Green (*Ca*. Nha. antarcticus) as described above (1 μM final concentration). Cells were then mixed and incubated at 30 °C. Samples (10 μL) were taken hourly and additionally stained with Concanavalin A (Alexa Fluor 350 conjugated, 200 μg/mL) and RedDot 2 (200× solution diluted to 1× final concentration). Cells were mounted onto glass slides and imaged on a Carl Zeiss Imager M.2 microscope at room temperature with a 100× Neofluor objective using a Carl Zeiss filter sets 02 (Ex: G 365 nm; Em: LP 420 nm), 38 (Ex: BP 470/40 nm; Em: BP 525/50 nm), 00 (Ex: BP 530–585 nm; Em: LP 615 nm), and 50 (Ex: BP 640/30 nm, Em: BP 690/50 nm). To assess the effects of MitoTracker dye on cell growth, *Hrr. lacusprofundi* R1S1 cells were stained with MitoTracker Orange (1 μM final concentration) as described above. MitoTracker-stained and unstained control cells were each inoculated into 5 mL fresh DBCM2 medium in 50 mL conical tubes (three biological replicates) to an optical density (OD_595_) of ~0.05, cultures incubated with shaking (150 RPM) at 30 °C, duplicate aliquots dispensed daily into microtitre plates, and OD_595_ monitored using a SpectraMax 190 Microplate Reader (Molecular Devices LLC) with fresh DBCM2 medium as a blank. To assess the effects of MitoTracker dye reversal on the interactions between *Ca*. Nha. antarcticus and *Hrr. lacusprofundi*, FACS-purified *Ca*. Nha. antarcticus cells were stained with MitoTracker Orange CMTMRos (1 μM final concentration), *Hrr. lacusprofundi* R1S1 cells were stained with MitoTracker Deep Red FM (1 μM final concentration), and cell mixtures were imaged using fluorescence time-lapse microscopy as described above. Reversing the labelling with dyes yielded analogous results to prior experiments (Supplementary Fig. 25). To assess the effects of cell fixation on interactions between *Ca*. Nha. antarcticus and *Hrr. lacusprofundi*, FACS-purified *Ca*. Nha. antarcticus cells (500 μL, ~2 × 10^7 ^mL^−1^) were pelleted (5 min, 19,745 *g*) and gently resuspended in 1 mL 18% buffered salt water^44^ containing 4 % (v/v) paraformaldehyde (PFA) and cells fixed by shaking (250 RPM) at room temperature overnight. The fixed cells were washed twice by centrifugation (5 min, 19,745 *g*), and the cell pellet resuspended in 500 μL DBCM2 medium. The fixed *Ca*. Nha. antarcticus cells were stained with MitoTracker Deep Red FM and incubated with *Hrr. lacusprofundi* R1S1 cells stained with MitoTracker Orange CMTMRos, and the cells imaged as described above. Pre-treatment of *Ca*. Nha. antarcticus cells with paraformaldehyde led to a reduced number of *Hrr. lacusprofundi* cells with attached *Ca*. Nha. antarcticus cells (106 of 186 imaged *Hrr. lacusprofundi* cells; 57%) and subsequently fewer lysed *Hrr. lacusprofundi* cells (12 cells; 6.5%) (Supplementary Fig. 25, Supplementary Dataset 1). Pre-treatment of the *Hrr. lacusprofundi* cells with paraformaldehyde also resulted in a substantial reduction in the frequency of both attachment (31 of 265 imaged *Hrr. lacusprofundi* cells; 11.7%) and lysis events (no cells: 0%) (Supplementary Fig. 25, Supplementary Dataset 1). Agarose pad time-course experiments were performed by staining *Hrr. lacusprofundi* R1S1 cells with MitoTracker Orange CMTMRos and FACS-purified *Ca*. Nha. antarcticus cells with MitoTracker Deep Red FM, as described above. The mixed cultures were sampled at different time points (0, 3, 6, 9, 12 and 24 h) and placed on a 1% (w/v) agarose pad containing DBCM2 basal salts on a glass slide with a #1.5 glass coverslip placed on top, and cells imaged as described above.

Microfluidic time-course interactions between *Ca*. Nha. antarcticus and *Hrr. lacusprofundi* were performed using a CellASIC ONIX2 microfluidics system to immobilise and record live cells that were exposed to a constant flow of liquid. CellASIC B04A plates (EMD Millipore) were equilibrated with 1 mg mL^−1^ Bovine Serum Albumin in phosphate-buffered saline followed by DBCM2 basal salts at a constant flow pressure of 5 psi. The mixed cell culture (*Hrr. lacusprofundi* R1S1 stained with MitoTracker Orange CMTMRos, and FACS-purified *Ca*. Nha. antarcticus cells stained with MitoTracker Deep Red FM) were loaded into the microfluidics chamber and perfused with DBCM2 medium at 0.25 psi for up to 48 h. Cells were imaged at 30 °C every hour or 30 min using a Nikon TiE2 inverted microscope fitted with a 100× oil-immersion phase-contrast NA 1.45 objective with TRITC (Ex: 561 nm; Em: 589–623 nm) and Cy5 (Ex: 640 nm; Em: 677–711 nm) filters.

For display purposes, time-lapse images were prepared by using OMERO and where needed adjusted for enhancing brightness with same setting applied to the whole image series. The quantitative analysis for the attachment, lysis, morphological change events and cell area were performed by combining automated detection (in FIJI 1.52P^45^ and Microbe J 5.13I^46^) and manual curation. Cell outlines were detected in MicrobeJ by phase-contrast image using the Local Default method and manually corrected where needed. Fluorescence signals were detected by “Maxima” in Microbe J using the Foci and Basic modes (*Hrr. lacusprofundi* fluorescence: tolerance 1000, Z score 20, area > 0.5 intensity > 800; *Ca*. Nha. antarcticus fluorescence: tolerance 1000, Z-score 6, area > 0.05, intensity > 200). For quantification of interactions in experiments using MitoTracker Green and Orange, Concanavalin A, and Reddot 2, channels were subjected to auto thresholding (Moments dark stack: MitoTracker Green, MitoTracker Orange, and Reddot 2; MaxEntropy dark stack: Concanavalin A). Channels were then converted to binary masks and particles counted (“Analyze Particles…”, “size=0.1-Infinity summarize in_situ”). Interactions between *Ca*. Nha. antarcticus and *Hrr. lacusprofundi* were quantified by taking overlaps between MitoTracker Green and MitoTracker Orange (ImageCalculator(“AND create”)) and counting particles (“Analyze Particles…”, “summarize in_situ”). Association of *Ca*. Nha. antarcticus with lysis events was quantified by taking overlaps between MitoTracker Green and Reddot 2 (ImageCalculator(“AND create”)) and counting particles (“Analyze Particles…”, “summarize in_situ”).

### 16S rRNA fluorescence in-situ hybridisation microscopy

For 16S rRNA-targeted FISH microscopy purified *Ca*. Nha. antarcticus cells were mixed with *Hrr. lacusprofundi* cells and incubated for 16 h, shaking (100 r.p.m.) at 30 °C. Following incubation cells were fixed in 2.5% glutaraldehyde overnight at 4 °C. Samples were pelleted (10 min, 20,000 *g*) and stained with 16 S rRNA specific probes (Table 1) as previously described^1,47^. Following 16S FISH probe hybridisation, samples were stained with ConA-AF350 as described above (see *Live fluorescence microscopy*). Samples were mounted onto glass slides and images acquired on an Eclipse Ti2 inverted microscope (Nikon) with a SoRa scanner (Yokogawa) and a Prime 95B sCMOS camera (Nikon). Imaging was performed with a 100× oil immersion objective (Plan Apo TIRF 100×/1.45, Nikon) using 2.8× magnification of the SoRa unit (effective total magnification of 280×). Z-stacks were acquired using a 200 ms exposure time with 10% laser power and 0.22 µm step size (15 slices, ~3.3 µm range).

**Table 1:** Details of 16S rRNA FISH probes

Name	Target Organism	Sequence
Hrr_FISH_Probe	*Hrr. lacusprofundi*	/5Cy3/TTATTACAGTCGACGCTGGTGAGATGTCCG
Nha_FISH_Probe	*Ca*. Nha. antarcticus	/5Cy5/GTGTATCCCAGAGCATTCG

### qPCR measurements

For qPCR-based measurements of 16S rRNA copy number organism specific 16S rRNA qPCR primers were used (Table 2). To produce standards for qPCR, PCR amplification of 16S rRNA gene fragments from both organisms was followed by cloning of fragments into pGEM-T easy vector (Promega) and transformation of JM109 competent cells (Promega) following manufacturer’s instructions. Plasmids were extracted using a peqGOLD Plasmid Miniprep Kit following manufacturer’s instructions, concentration measured, and serial dilutions carried out. qPCR reactions were carried out using a CFX96 Real-Time PCR Detection System (Bio-Rad) for 40 cycles with an annealing temperature of 55 °C.

**Table 2:** Details of qPCR primers

Name	Target Organism	Sequence
H_lac_qpcrF	*Hrr. lacusprofundi*	GGATTGTGCCAAAAGCTCCG
H_lac_qpcrR	*Hrr. lacusprofundi*	ACTCTCATGACCCGTACCGA
Nanohalo_qpcrF	*Ca*. Nha. antarcticus	ACTTAAAGGAATTGACGGGGG
Nanohalo_qpcrR	*Ca*. Nha. antarcticus	CATGCAGCTCCTCTCAGCG

### Cryo-electron microscopy and tomography

For cryo-CLEM, purified *Ca*. Nha. antarcticus and *Hrr. lacusprofundi* cells were stained as described (see *Live fluorescence microscopy*), mixed at a cell-to-cell ratio of 1:3 and incubated for a period of 16 h. Samples were screened using a Zeiss Imager M2 widefield microscope using the ZenBlue software (Carl Zeiss AG) in order to assess staining. Once screened, the sample was loaded onto Quantifoil holey carbon coated grids (Au/3.5/1 200 mesh; Quantifoil Micro Tools, Jena, Germany) and cryo-fixed by plunge freezing with a Leica EM GP2 (Leica Microsystems) into liquid ethane and stored in liquid nitrogen until imaging, as previously described^48,49^. Grids were assembled into autogrids (ThermoFisher) and imaged using a Zeiss LSM 900 upright confocal with Airyscan 2 fluorescence microscope on a Linkam CMS196V3 cryo-correlative microscopy stage with a 100× NA 0.75 objective using a FITC filter at an excitation of 488 nm for MitoTracker Green and a Cy5 filter at an excitation of 647 nm for MitoTracker DeepRed. Images were acquired using an Axiocam 506 mono camera (Carl Zeiss AG), as previously described^50,51^. Autogrids were transferred for imaging to a Titan Krios G3 (ThermoFisher) cryo-electron microscope operating at 300 kV equipped with a Bioquantum energy filter (slit width 20 eV) and the K3 detector (Gatan Inc.). For cryo-ET of fluorescently labelled and internalised *Ca*. Nha. Antarcticus derived structures, tilt series were collected dose-symmetrically^52^, with 3° increments between ±60° at a pixel size of 3.37 Å/pixel using SerialEM^53^. The defocus range of the tilt-series was varied between −5 to −8 µm and a total dose of ~80e^−^/Å^2^ was applied over the entire series, corresponding to ~1.95e^−^/Å^2^ per tilt-image which were dose-fractioned into six frames.

For cryo-ET of *Hrr. lacusprofundi*—*Ca*. Nha. antarcticus co-cultures without correlated fluorescence microscopy, cells were mixed as described above for fluorescence microscopy and incubated at 30 °C for 17 h. Cells were then loaded onto Quantifoil holey carbon coated grids (Cu/Rh 3.5/1 200 mesh for *Hrr. lacusprofundi* cells and co-cultures and Cu/Rh 2/2 200 mesh for the pure *Ca*. Nha. antarcticus cells). Samples were cryo-fixed by plunge-freezing in liquid ethane using a Vitrobot Mark IV and stored under liquid nitrogen until imaging, as previously described^48,49^. Imaging was performed on a Titan Krios G3 at 300 kV using a Bioquantum energy filter and the K3 detector (Gatan Inc.). Tilt series were collected at 2° increments between ±60°, defocus was varied from −6 to −12 µm depending on the tilt series (specified in figure legends), and a total dose of 80e^−^/Å^2^ was applied over the series. Tilt-movies were motion aligned using the *alignframes* programme implemented in IMOD^54^ while additionally saving odd and even motion-aligned tilt-series. All tomographic alignments and reconstructions were performed using IMOD^54^ (fluorescent stained samples) and tomo3D^55^ (non-stained samples). IMOD reconstructed tomograms were additionally denoised with Cryo-CARE^56,57^ using the odd and even motion-aligned tilt-series. Fluorescence micrographs and cryo-TEM images were manually aligned to generate overlays during figure production.

### Bioinformatic analyses

For analysing groups of orthologous proteins, a list of all archaeal genomes was downloaded from NCBI. Genomes were filtered on the basis of stage of assembly (scaffold) and number of scaffolds (<100) in order to produce a reduced list of moderate quality genomes for analysis (607 total). Nucleotide and amino acid fasta files were downloaded for those genomes using a custom python script through the NCBI ftp site. Protein sequences from the genomes were run through Orthofinder 2.3.1^58^ using the diamond blast option (-t 16 -S diamond) in order to identify orthologous proteins shared amongst the genomes. Once orthogroups had been identified they were filtered using a custom python script to identify groups unique to DPANN. These orthogroups were then subjected to preliminary domain annotation using InterProScan version 5.25-64.0^59^.

For the identification of proteins potentially involved in cell-cell interactions, initial analyses of DPANN-specific protein clusters revealed that many DPANN seemingly encoded one or two proteins with putative nucleopore domains that possessed predicted coiled-coil structures. To investigate these proteins in more detail and determine their distribution across Archaea, HMM profiles were generated from these coiled-coil protein (CCP) amino acid sequences in DPANN and the profiles were used as queries against an archaeal reference database (569 species, Supplementary Dataset 12). Specifically, the protein sequences from the relevant orthogroups were aligned using MAFFT L-INS-I v7.407^60^ and trimmed using BMGE v1.12 (settings: -t AA -m BLOSUM30 -h 0.55)^61^. Subsequently, protein domains were predicted using HHpred within the hh-suite^62^ with the following two steps: hhblits v3.3.0 was run (settings: -i trimmed_alignment -E 1E-01 -d uniclust30_2018_08) and provided the input a3m file for hhsearch v3.1b2 (settings: -i a3m_file -d pdb70 -p 20 -Z 250 -loc -z 1 -b 1 -B 250 -ssm 2 -sc 1 -seq 1 -dbstrlen 10000 -norealign -maxres 32000 -contxt context_data.crf -blasttab). The top 50 hits were manually inspected for a match to a potential nucleopore domain. The exact positions of the domain in the respective proteins were extracted from the full protein alignment using bedtools v2.26.0^63^ and used to build HMM profiles with hmmbuild. The two profiles were used to search for potential nucleopore domain proteins across our archaeal reference database using a custom script (hmmsearchTable) which implements the hmmsearch algorithm (available in 3_Scripts.tar.gz at https://zenodo.org/record/3839790#.Xywn7btR23U). In order to annotate the identified proteins and verify the presence of nucleopore domains, positive hits were extracted and analysed with HHpred. The potential secondary and tertiary structures of the positive hits were also examined by investigating the protein sequences using the Phyre2 webserver^64^. Additionally, the secondary domain structure of these proteins from *Ca*. Nha. antarcticus was investigated using JPred4^65^. The protein sequences of the ten genes up- and downstream surrounding the CCP genes were examined and all annotations (see below) are provided (Supplementary Dataset 13), including the top hits of the HHpred and Phyre2 results for the CCPs. To complement Phyre2 predictions OmegaFold^66^ structural predictions were produced for all predicted coding sequences in the *Ca*. Nha. antarcticus genome. Structurally similar proteins were identified using FoldSeek^67^ and results summarised in Supplementary Dataset 5.

In order to ensure consistency, all genomes were annotated using the same settings and databases. Gene calling was performed using Prokka^68^ (v1.14, settings: –kingdom Archaea –addgenes –increment 10 –compliant –centre UU –norrna –notrna). For further functional annotation, the generated protein files were compared against several databases, including the arCOGs (version from 2014)^69^, the KO profiles from the KEGG Automatic Annotation Server (KAAS; downloaded April 2019)^70^, the Pfam database (Release 31.0)^71^, the TIGRFAM database (Release 15.0)^72^, the Carbohydrate-Active enZymes (CAZy) database (downloaded from dbCAN2 in September 2019)^73^, the MEROPs database (Release 12.0)^74^, the Transporter Classification Database (TCDB; downloaded in November 2018)^75^, the hydrogenase database (HydDB; downloaded in November 2018)^76^ and NCBI_nr (downloaded in November 2018). Additionally, all proteins were scanned for protein domains using InterProScan (v5.29-68.0; settings: –iprlookup –goterms)^59^. ArCOGs were assigned using PSI-BLAST v2.7.1+ (settings: -evalue 1e-4 -show_gis -outfmt 6 -max_target_seqs 1000 -dbsize 100000000 -comp_based_stats F -seg no)^77^. KOs as well as PFAMs, TIGRFAMs and CAZymes were identified in all archaeal genomes using hmmsearch v3.1b298 (settings: -E 1e-4)^78^. The Merops database was searched using BLASTp v2.7.1 (settings: -outfmt 6, -evalue 1e-20)^74^. For all database searches, the best hit for each protein was selected based on the highest e-value and bitscore. For InterProScan, multiple hits corresponding to the individual domains of a protein were reported using a custom script (parse_IPRdomains_vs2_GO_2.py).

In order to identify genes that may be involved in the internalisation process, an all-vs-all BLAST (BLASTp v2.7.1 settings: -outfmt 6, -evalue 1e-30)^77^ was performed on the *Ca*. Nha. antarcticus genome against the *Ca*. Nanohalobium genome (Supplementary Dataset 14). Hits that aligned to <30% of the reference sequence were discarded. *Ca*. Nha antarcticus genes that did not have an identified homologue in *Ca*. Nanohalobium were then subjected to structural prediction through the Phyre2 server (Supplementary Dataset 4)^64^. Structural predictions were reviewed alongside functional annotations (Supplementary Datasets 3−5) in order to assess likelihood of involvement in the internalisation process.

### Phylogenetic analyses

Maximum likelihood phylogenetic reconstructions of an archaeal species tree were performed using a combination of the GDTB^79^, ribosomal^7^ and phylosift^80^ marker sets. Briefly, using hmmsearch, a modified TIGRFAM database was queried against a protein database generated from proteins called from 569 archaeal species^8^ (custom scripts are available at https://zenodo.org/record/3839790#.Xywn7btR23U). An initial set of 151 marker protein trees was manually investigated for resolved monophyletic clades of well-established archaeal phylum- or order-level lineages resulting in 51 marker proteins (Supplementary Dataset 15) used for further analyses. In particular, the 151 single gene trees were generated by individually aligning marker proteins using MAFFT L-INS-i v7.407 (settings: –reorder)^60^, trimming using BMGE v1.12 (settings: -t AA -m BLOSUM30 -h 0.55)^61^ and inferring phylogenetic trees using IQ-TREE (v1.6.7, settings: -m LG + G -wbtl -bb 1000 -bnni)^81^. After selecting the final marker set, the 51 non-redundant marker proteins of interest were extracted from the larger database and individually aligned using MAFFT L-INS-i v7.407 (settings: –reorder)^60^ and trimmed using BMGE v1.12 (settings: -t AA -m BLOSUM30 -h 0.55)^61^. The single proteins were concatenated using catfasta2phyml.pl (https://github.com/nylander/catfasta2phyml) and a phylogenetic tree was generated using IQ-TREE (v1.6.7, settings: -m LG + C20 + F + R -bb 1000 -alrt 1000)^81^, visualised using FigTree (v1.4.4) and annotated with Inkscape and Adobe Illustrator.

Single protein trees for relevant pili genes were generated as follows: Pili proteins were identified and extracted based on their arCOG identifiers from the archaeal reference set and a bacterial reference database (3022 species, Supplementary Dataset 17). arCOGs belonging to the same COG were combined (see Supplementary Dataset 20). Single gene trees were generated by individually aligning marker proteins using MAFFT L-INS-i v7.407 (settings: –reorder)^60^, trimming using BMGE v1.12 (settings: -t AA -m BLOSUM30 -h 0.55)^61^ and inferring phylogenetic trees using IQ-TREE (v1.6.7, settings: -m LG + C10 + F + R -nt 5 -wbtl -bb 1000 -bnni)^81^.

### Reporting summary

Further information on research design is available in the Nature Portfolio Reporting Summary linked to this article.

### Supplementary information

Supplementary Information

Reporting Summary

Description of Additional Supplementary Files

Peer Review File

Supplementary Datasets 1 - 20

Supplementary Videos 1 - 17

### Source data

Source Data

## Data Availability

Supplementary data including hi-resolution versions of main text figures and Supplementary Movies have been deposited in our repository on figshare [10.6084/m9.figshare.12957092.v1]. All datasets generated and/or analysed during this study are available in our data repository at Zenodo [10.5281/zenodo.4707105]. All quantitative data for cultures and bioinformatics data are provided in the Supplementary Dataset and Source Data files. Public databases used in this study are the following: the arCOG database (version from 2014) downloaded from [ftp://ftp.ncbi.nih.gov/pub/wolf/COGs/arCOG/], the KO profiles downloaded from the KEGG Automatic Annotation Server in April 2019 [https://www.genome.jp/tools/kofamkoala/], the Pfam database (Release 31.0) [ftp://ftp.ebi.ac.uk/pub/databases/Pfam/releases/], the TIGRFAM database (Release 15.0) [ftp://ftp.jcvi.org/pub/data/TIGRFAMs/], the Carbohydrate-Active enZymes (CAZy) database downloaded from dbCAN2 in September 2019 [http://bcb.unl.edu/dbCAN2/download/], the MEROPs database (Release 12.0) [https://www.ebi.ac.uk/merops/download_list.shtml], the Transporter Classification Database(TCDB) downloaded in November 2018 [http://www.tcdb.org/download.php], the hydrogenase database (HydDB) downloaded in November 2018 [https://services.birc.au.dk/hyddb/browser/], and NCBI_nr downloaded in November 2018 [ftp://ftp.ncbi.nlm.nih.gov/blast/db/]. Source data are provided with this paper.

## Code availability

All custom scripts and workflows used to generate data can be found in our data repository on zenodo [10.5281/zenodo.4707105].
