# Supplementary material for: The parasitic lifestyle of an archaeal symbiont
Source: Nat Commun. 2024 Jul 31;15:6449. doi: 10.1038/s41467-024-49962-y (PMC11291902; doi:10.1038/s41467-024-49962-y)
Supplement: Supplementary file 3 — Description of Additional Supplementary Files [file 41467_2024_49962_MOESM3_ESM.pdf]

Description of Additional Supplementary Files:

Supplementary Dataset 1: Quantitative data for observed lysis events and morphological changes during live fluorescence experiments. Co-culture experiments displayed consistently higher rates of Hrr. lacusprofundi cell lysis and morphological changes than pure Hrr. lacusprofundi controls. nd: not determined, N/A: not applicable

Supplementary Dataset 2: Quantitative data for attachment and lysis dynamics over short term live fluorescence experiments (n=3). Ca. Nha. antarcticus cells rapidly attached to Hrr. lacusprofundi cells following mixing of the two organisms. Over the course of the time series the number of Hrr. lacusprofundi lysis events associated with Ca. Nha. antarcticus cells increased steadily.

Supplementary Dataset 3: Annotation table used in this study to characterize Ca. Nha. antarcticus. Annotations were done using several databases as shown (General protein annotations). The blast results refer to the blastp search against ncbi_nr. Ca. Nanohalobium homolog indicates whether a homolog was identified in the Ca. Nanohalobium MAG and if so what the ID of that protein is.

Supplementary Dataset 4: Phyre2 structural predictions of Ca. Nha. antarcticus proteins that did not have identifiable homologs in the Ca. Nanohalobium MAG

Supplementary Dataset 5: FoldSeek search output for OmegaFold structural predictions of Ca. Nha. antarcticus proteins

Supplementary Dataset 6: List of domains annotated by InterProScan 5.25-64.0 on DPANN unique orthologous groups of proteins and the number of protein families annotated with each domain.

Supplementary Dataset 7: List of InterProScan 5.25-64.0 annotations for each group of orthologous proteins unique to DPANN Archaea.

Supplementary Dataset 8: HHPred annotations of full length DPANN proteins predicted to possess nucleoporin-like domains.

Supplementary Dataset 9: HHPred annotation of subregions predicted to have similarity to nucleoporins of DPANN proteins presented in Supplementary Dataset 5.

Supplementary Dataset 10: Phyre2 annotations of full length DPANN proteins predicted to possess nucleoporin-like domains.

Supplementary Dataset 11: Phyre2 annotation of subregions predicted to have similarity to nucleoporins of DPANN proteins presented in Supplementary Dataset 7.

Supplementary Dataset 12: Taxonomy information for the genomes used to generate Fig. 4. Lists the NCBI accession ID as well as all relevant information to download the data from NCBI as well as the full taxonomy string.

Supplementary Dataset 13: List of Locus 1 and Locus 2 CCP homologs detected across a set of 569 archaeal species as well as the 10 proteins upstream and downstream. Summarizes the HHphred results for the CCPs by summarizing the top hit (HHpred Top hit), the hit for Nup155 (HHpred Nup155 hit) and the hit for a potential capsid protein (HHpred capsid hit) including the hit number. Additionally, the top PHYRE2 results for CCPs are shown (PHYRE2 results (Top hit)). For the full locus a full list of annotations using several databases is shown as well (General protein annotations). To search for relevant hits to Ca. Nanohaloarchaeum antarcticus one can search for N_antarcticus in the BinID column.

Supplementary Dataset 14: All-vs-All BLAST of Ca. Nha. antarcticus predicted ORFs against the Ca. Nanohalobium MAG. Hits were subsequently filtered on the basis of e-value (1e-30) and percentage of sequence aligned (>30%)

Supplementary Dataset 15: Marker genes used for concatenated phylogenetic analyses. Origin: The original database proteins were recovered from. Ogs: Orthologous group marker proteins recovered from Dombrowski et al., 2020a; 122 GDTB: 122 archaeal GTDB marker set recovered from the GTDB marker set was downloaded February 2019 (release r86 downloaded from [https://gtdb.ecogenomic.org] and is comprised of 122 HMM profiles from the PFAM and TIGRFAM databases.

Supplementary Dataset 16: Taxonomy information for the genomes used to generate Pilus gene trees. Lists the NCBI accession ID as well as all relevant information to download the data from NCBI as well as the full taxonomy string.

Supplementary Dataset 17: Pili related proteins found across investigated archaeal genomes (summarized in Supplementary Dataset 13). Summarized on phylum to order level. Parentheses: Total number of genomes investigated per clade. Table was generated based on Makarova et al., 2016. arCOGs highlighted in dark green were identified within DPANN operons.

Proteins highlighted in light green were not identified in DPANN operons but were discussed elsewhere.

Supplementary Dataset 18: Top 50 HHphred results for CCPs detected across 569 archaeal species. For detailed information of the annotations on each protein, see Supplementary Dataset 10 for details. Green: Hits to potential nucleopore proteins, yellow: hits to potential capsid proteins. To search for relevant hits to Ca. Nha antarcticus one can search for N_antarcticus in the BinID column.

Supplementary Dataset 19: Top 20 PHYRE2 results for CCPs detected across 569 archaeal species. For detailed information of the annotations on each protein, see Supplementary Table 10. To search for relevant hits to Ca. Nha antarcticus, search for N_antarcticus in the BinID column.

Supplementary Dataset 20: Pili related proteins found across investigated archaeal genomes. Summarized on phylum to order level. Parentheses: Total number of genomes investigated per clade. Table was generated based on Makarova et al., 2016a. arCOGs highlighted in green were discussed in the manuscript.

Supplementary Movie 1: Live cell imaging of microfluidics cultivation experiments. Movie shows phase-contrast time lapse of a Hrr. lacusprofundi and Ca. Nha. antarcticus coculture (left panel) and a pure Hrr. lacusprofundi culture (right panel) grown in microfluidics chambers at 30 °C. Images were acquired on a Nikon TiE2 inverted microscope fitted with a 100× oil immersion phase-contrast NA 1.45 objective once per hour for a period of 21 hours.

Supplementary Movie 2: Cryo-ET of Hrr. lacusprofundi and Ca. Nha antarcticus coculture imaged with cryo-CLEM. Movie shows a cryo-electron tomogram of a Hrr. lacusprofundi cell with a Ca. Nha. antarcticus cell visible in the cytoplasm (Z-slice from this tomogram is shown in Fig. 3d and e). Tilt series was acquired on a Titan Krios at 300 kV.

Supplementary Movie 3: Cryo-ET of Hrr. lacusprofundi and Ca. Nha antarcticus coculture imaged with cryo-CLEM. Movie shows a cryo-electron tomogram of a Hrr. lacusprofundi cell with a Ca. Nha. antarcticus cell visible in the cytoplasm (Z-slice from this tomogram is shown in Supplementary Fig. 11d and e). Tilt series was acquired on a Titan Krios at 300 kV.

Supplementary Movie 4: Cryo-ET of Hrr. lacusprofundi and Ca. Nha antarcticus coculture. Movie shows a cryo-electron tomogram of a Hrr. lacusprofundi cell with internal structures consistent with Ca. Nha. antarcticus cells visible in the cytoplasm (Zslice from this tomogram is shown in Supplementary Fig. 12a and b). Tilt series was acquired on a Titan Krios at 300 kV.

Supplementary Movie 5: Cryo-ET of Hrr. lacusprofundi and Ca. Nha antarcticus coculture. Movie shows a cryo-electron tomogram of a Hrr. lacusprofundi cell with an internal structure consistent with a Ca. Nha. antarcticus cell visible in the cytoplasm (Z-slice from this tomogram is shown in Supplementary Fig. 12c). Tilt series was acquired on a Titan Krios at 300 kV.

Supplementary Movie 6: Cryo-ET of Hrr. lacusprofundi and Ca. Nha antarcticus coculture. Movie shows a cryo-electron tomogram of a Hrr. lacusprofundi cell with internal structures consistent with Ca. Nha. antarcticus cells visible in the cytoplasm. Tomogram also shows a Ca. Nha. antarcticus cell that appears to be in the process of internalising within the Hrr. lacusprofundi cell (Z-slice from this tomogram is shown in Supplementary Fig. 12d - f). Tilt series was acquired on a Titan Krios at 300 kV.

Supplementary Movie 7: Cryo-ET of Hrr. lacusprofundi and Ca. Nha antarcticus coculture. Movie shows a cryo-electron tomogram of a Hrr. lacusprofundi cell that has lysed with an internal structure consistent with a Ca. Nha. antarcticus cell visible in the cytoplasm (Z-slice from this tomogram is shown in Supplementary Fig. 13a and b). Tilt series was acquired on a Titan Krios at 300 kV.

Supplementary Movie 8: Cryo-ET of Hrr. lacusprofundi and Ca. Nha antarcticus coculture. Movie shows a cryo-electron tomogram of a Hrr. lacusprofundi cell with internal structures consistent with Ca. Nha. antarcticus cells visible in the cytoplasm (Zslice from this tomogram is shown in Supplementary Fig. 13c and d). Tilt series was acquired on a Titan Krios at 300 kV.

Supplementary Movie 9: Cryo-ET of a pure Ca. Nha antarcticus sample. Movie shows a cryoelectron tomogram of a purified Ca. Nha. antarcticus cell used for co-culture experiments. Tomogram shows presence of internal structures resembling membrane embedded lipid droplets and PHA-like granules (Z-slice from this tomogram is shown in Supplementary Fig. 14a). Tilt series was acquired on a Titan Krios at 300 kV.

Supplementary Movie 10: Cryo-ET of a pure Ca. Nha antarcticus sample. Movie shows acryo-electron tomogram of a purified Ca. Nha. antarcticus cell used for co-culture experiments. Tomogram shows presence of internal structures resembling membrane embedded lipid droplets and PHA-like granules (Z-slice from this tomogram is shown in Supplementary Fig. 14b). Tilt series was acquired on a Titan Krios at 300 kV.

Supplementary Movie 11: Cryo-ET of a pure Ca. Nha antarcticus sample. Movie shows a cryo-electron tomogram of a purified Ca. Nha. antarcticus cell used for co-culture experiments. Tomogram shows presence of internal structures resembling membrane embedded lipid

droplets and PHA-like granules (Z-slice from this tomogram is shown in Supplementary Fig. 14c). Tilt series was acquired on a Titan Krios at 300 kV.

Supplementary Movie 12: Cryo-ET of a pure Ca. Nha antarcticus sample. Movie shows a cryo-electron tomogram of a purified Ca. Nha. antarcticus cell used for co-culture experiments. Tomogram shows presence of internal structures resembling membrane embedded lipid droplets and PHA-like granules (Z-slice from this tomogram is shown in Supplementary Fig. 14d). Tilt series was acquired on a Titan Krios at 300 kV

Supplementary Movie 13: Cryo-ET of Hrr. lacusprofundi and Ca. Nha antarcticus coculture. Movie shows a cryo-electron tomogram of a Hrr. lacusprofundi cell interacting with a Ca. Nha. antarcticus cell showing membrane disruption at the contact site between the two cells (Z-slice from this tomogram is shown in Supplementary Fig. 14e and f). Tilt series was acquired on a Titan Krios at 300 kV.

Supplementary Movie 14: Cryo-ET of Hrr. lacusprofundi and Ca. Nha antarcticus coculture. Movie shows a cryo-electron tomogram of a Hrr. lacusprofundi cell interacting with a Ca. Nha. antarcticus cell showing membrane disruption at the contact site between the two cells (Z-slice from this tomogram is shown in Supplementary Fig. 14g and h). Tilt series was acquired on a Titan Krios at 300 kV.

Supplementary Movie 15: Cryo-ET of a pure Hrr. lacusprofundi culture. Movie shows a cryoelectron tomogram of a Hrr. lacusprofundi cell in pure culture showing no sign of internal structures similar to those seen in co-cultures (Z-slice from this tomogram is shown in Supplementary Fig. 14i). Tilt series was acquired on a Titan Krios at 300 kV.

Supplementary Movie 16: Cryo-ET of a pure Hrr. lacusprofundi culture. Movie shows a cryoelectron tomogram of a Hrr. lacusprofundi cell in pure culture showing no sign of internal structures similar to those seen in co-cultures (Z-slice from this tomogram is shown in Supplementary Fig. 14j). Tilt series was acquired on a Titan Krios at 300 kV

Supplementary Movie 17: Cryo-ET of a pure Hrr. lacusprofundi culture. Movie shows a cryoelectron tomogram of a Hrr. lacusprofundi cell in pure culture showing no sign of internal structures similar to those seen in co-cultures (Z-slice from this tomogram is shown in Supplementary Fig. 14k). Tilt series was acquired on a Titan Krios at 300 kV.
