# Supplementary material for: The parasitic lifestyle of an archaeal symbiont
Source: Nat Commun. 2024 Jul 31;15:6449. doi: 10.1038/s41467-024-49962-y (PMC11291902; doi:10.1038/s41467-024-49962-y)
Supplement: Supplementary file 2 — Reporting Summary [file 41467_2024_49962_MOESM2_ESM.pdf]

nature portfolio

Corresponding author(s): Joshua Norman Hamm

Last updated by author(s): Jun 3, 2024

Reporting Summary

Nature Portfolio wishes to improve the reproducibility of the work that we publish. This form provides structure for consistency and transparency in reporting. For further information on Nature Portfolio policies, see our Editorial Policies and the Editorial Policy Checklist.

Statistics

For all statistical analyses, confirm that the following items are present in the figure legend, table legend, main text, or Methods section.

n/a	Confirmed	
☒	☐	The exact sample size (n) for each experimental group/condition, given as a discrete number and unit of measurement
☒	☐	A statement on whether measurements were taken from distinct samples or whether the same sample was measured repeatedly
☒	☐	The statistical test(s) used AND whether they are one- or two-sided<br>*Only common tests should be described solely by name; describe more complex techniques in the Methods section.*
☒	☐	A description of all covariates tested
☒	☐	A description of any assumptions or corrections, such as tests of normality and adjustment for multiple comparisons
☒	☐	A full description of the statistical parameters including central tendency (e.g. means) or other basic estimates (e.g. regression coefficient) AND variation (e.g. standard deviation) or associated estimates of uncertainty (e.g. confidence intervals)
☒	☐	For null hypothesis testing, the test statistic (e.g. F, t, r) with confidence intervals, effect sizes, degrees of freedom and P value noted<br>*Give P values as exact values whenever suitable.*
☒	☐	For Bayesian analysis, information on the choice of priors and Markov chain Monte Carlo settings
☒	☐	For hierarchical and complex designs, identification of the appropriate level for tests and full reporting of outcomes
☒	☐	Estimates of effect sizes (e.g. Cohen's d, Pearson's r), indicating how they were calculated

Our web collection on statistics for biologists contains articles on many of the points above.

Software and code

Policy information about availability of computer code

Data collection	Fluorescence Microscopy data was acquired using NIS Elements (Nikon instruments), Zeiss Zen Blue (Carl Zeiss Instruments), and DigitalMicrograph (Cryo-Electron Microscopy. qPCR data was collected with BioRad Maestro software.
Data analysis	All custom scripts and workflows used to generate data can be found in our data repository on zenodo [https://doi.org/10.5281/zenodo.4707105].<br>Software used for Microscopy analyses were:<br>Imaris 10.1<br>ImageJ v1.53<br>Software used for bioinformatics analyses were:<br>Orthofinder 2.3.1<br>InterProScan version 5.25-64.0<br>MAFFT L-INS-I v7.407<br>BMGE v1.12<br>hhblits v3.3.0<br>hhsearch v3.1b2<br>bedtools v2.26.0<br>Phyre2 Webserver v2.0<br>JPred v4<br>OmegaFold v1<br>FoldSeek v1

Prokka v1.14
PSI-BLAST v2.7.1+
hmmsearch v3.1b298
BLASTp v2.7.1
IQ-TREE v1.6.7
FigTree v1.4.4

For manuscripts utilizing custom algorithms or software that are central to the research but not yet described in published literature, software must be made available to editors and reviewers. We strongly encourage code deposition in a community repository (e.g. GitHub). See the Nature Portfolio guidelines for submitting code & software for further information.

Data

Policy information about availability of data

All manuscripts must include a data availability statement. This statement should provide the following information, where applicable:

- Accession codes, unique identifiers, or web links for publicly available datasets
- A description of any restrictions on data availability
- For clinical datasets or third party data, please ensure that the statement adheres to our policy

Supplementary data including hi-resolution versions of main text figures and Supplementary Movies are availablehave been deposited in our repository on figshare [https://doi.org/10.6084/m9.figshare.12957092.v1]. All datasets generated and/or analysed during this study are available in our data repository at Zenodo [https://doi.org/10.5281/zenodo.4707105]. All quantitative data for cultures and bioinformatics data are provided in the Supplementary Dataset and Source Data files. Public databases used in this study are the following: the arCOG database (version from 2014) downloaded from [ftp://ftp.ncbi.nih.gov/pub/wolf/COGs/arCOG/], the KO profiles downloaded from the KEGG Automatic Annotation Server in April 2019 [https://www.genome.jp/tools/kofamkoala/], the Pfam database (Release 31.0) [ftp://ftp.ebi.ac.uk/pub/databases/Pfam/releases/], the TIGRFAM database (Release 15.0) [ftp://ftp.jcvi.org/pub/data/TIGRFAMs/], the Carbohydrate-Active enZymes (CAZy) database downloaded from dbCAN2 in September 2019 [http://bcb.unl.edu/dbCAN2/download/], the MEROPs database (Release 12.0) [https://www.ebi.ac.uk/merops/download_list.shtml], the Transporter Classification Database(TCDB) downloaded in November 2018 [http://www.tcdb.org/download.php], the hydrogenase database (HydDB) downloaded in November 2018 [https://services.birc.au.dk/hyddb/browser/], and NCBI_nr downloaded in November 2018 [ftp://ftp.ncbi.nlm.nih.gov/blast/db/].

Research involving human participants, their data, or biological material

Policy information about studies with human participants or human data. See also policy information about sex, gender (identity/presentation), and sexual orientation and race, ethnicity and racism.

Reporting on sex and gender	This research did not involve any human participants or human data.
Reporting on race, ethnicity, or other socially relevant groupings	This research did not involve any human participants or human data.
Population characteristics	This research did not involve any human participants or human data.
Recruitment	This research did not involve any human participants or human data.
Ethics oversight	This research did not involve any human participants or human data.

Note that full information on the approval of the study protocol must also be provided in the manuscript.

Field-specific reporting

Please select the one below that is the best fit for your research. If you are not sure, read the appropriate sections before making your selection.

[x] Life sciences   [ ] Behavioural & social sciences   [ ] Ecological, evolutionary & environmental sciences

For a reference copy of the document with all sections, see nature.com/documents/nr-reporting-summary-flat.pdf

Life sciences study design

All studies must disclose on these points even when the disclosure is negative.

Sample size	No statistical tests were performed and thus no tests on sample size were carried out.
Data exclusions	No data were excluded from the analyses.
Replication	All attempts to replicate experiments were successful. Live fluorescence experiments were carried out multiple times in 2 different labs with comparable results each time. Electron microscopy was carried out multiple times in 2 distinct facilities with comparable results each time.
Randomization	No randomization was performed. All experiments were conducted using microbial cultures containing genetically identical strains.
Blinding	Investigators were not blinded. Data acquisition was carried out automatically by microscopy instruments and analysed using established pipelines for both experimental and control conditions. Only once processed images were finalised did investigators view the results.

Reporting for specific materials, systems and methods

We require information from authors about some types of materials, experimental systems and methods used in many studies. Here, indicate whether each material, system or method listed is relevant to your study. If you are not sure if a list item applies to your research, read the appropriate section before selecting a response.

Materials & experimental systems

n/a	Involved in the study
☒	☐ Antibodies
☒	☐ Eukaryotic cell lines
☒	☐ Palaeontology and archaeology
☒	☐ Animals and other organisms
☒	☐ Clinical data
☒	☐ Dual use research of concern
☒	☐ Plants

Methods

n/a	Involved in the study
☒	☐ ChIP-seq
☐	☒ Flow cytometry
☒	☐ MRI-based neuroimaging

Plants

Seed stocks

Report on the source of all seed stocks or other plant material used. If applicable, state the seed stock centre and catalogue number. If plant specimens were collected from the field, describe the collection location, date and sampling procedures.

Novel plant genotypes

Describe the methods by which all novel plant genotypes were produced. This includes those generated by transgenic approaches, gene editing, chemical/radiation-based mutagenesis and hybridization. For transgenic lines, describe the transformation method, the number of independent lines analyzed and the generation upon which experiments were performed. For gene-edited lines, describe the editor used, the endogenous sequence targeted for editing, the targeting guide RNA sequence (if applicable) and how the editor was applied.

Authentication

Describe any authentication procedures for each seed stock used or novel genotype generated. Describe any experiments used to assess the effect of a mutation and, where applicable, how potential secondary effects (e.g. second site T-DNA insertions, mosiacism, off-target gene editing) were examined.

Flow Cytometry

Plots

Confirm that:

☒ The axis labels state the marker and fluorochrome used (e.g. CD4-FITC).

☒ The axis scales are clearly visible. Include numbers along axes only for bottom left plot of group (a 'group' is an analysis of identical markers).

☒ All plots are contour plots with outliers or pseudocolor plots.

☒ A numerical value for number of cells or percentage (with statistics) is provided.

Methodology

Sample preparation	Cultures were grown to stationary phase and diluted with fresh media immediately prior to cytometry.
Instrument	BDInflux
Software	BD FACS™ Sortware sorter software
Cell population abundance	Post sort fractions were assessed for purity with PCR targeting Ca. Nha. antarcticus and Hrr. lacusprofundi. Positive amplification for Ca. Nha. antarcticus and negative for Hrr. lacusprofundi was required for sorting to be considered successful.
Gating strategy	Gating was performed on the basis of FSC vs SSC to select for cell size. Standard size beads were used to calibrate size estimates and set gate bounds.

☒ Tick this box to confirm that a figure exemplifying the gating strategy is provided in the Supplementary Information.
